# Early Infantile Epileptic Encephalopathy in an* STXBP1* Patient with Lactic Acidemia and Normal Mitochondrial Respiratory Chain Function

**DOI:** 10.1155/2016/4140780

**Published:** 2016-03-16

**Authors:** Dong Li, Elizabeth Bhoj, Elizabeth McCormick, Fengxiang Wang, James Snyder, Tiancheng Wang, Yan Zhao, Cecilia Kim, Rosetta Chiavacci, Lifeng Tian, Marni J. Falk, Hakon Hakonarson

**Affiliations:** ^1^Center for Applied Genomics, The Children's Hospital of Philadelphia, Philadelphia, PA 19104, USA; ^2^Division of Human Genetics, The Children's Hospital of Philadelphia, Philadelphia, PA 19104, USA; ^3^Division of Metabolic Disease, Department of Pediatrics, The Children's Hospital of Philadelphia and the Perelman School of Medicine, Philadelphia, PA 19104, USA

## Abstract

A wide range of clinical findings have been associated with mutations in Syntaxin Binding Protein 1 (*STXBP1*), including multiple forms of epilepsy, nonsyndromic intellectual disability, and movement disorders.* STXBP1* mutations have recently been associated with mitochondrial pathology, although it remains unclear if this phenotype is a part of the core feature for this gene disorder. We report a 7-year-old boy who presented for diagnostic evaluation of intractable epilepsy, episodic ataxia, resting tremor, and speech regression following a period of apparently normal early development. Mild lactic acidemia was detected on one occasion at the time of an intercurrent illness. Due to the concern for mitochondrial disease, ophthalmologic evaluation was performed that revealed bilateral midperiphery pigmentary mottling. Optical coherence tomography (OCT) testing demonstrated a bilaterally thickened ganglion cell layer in the perifovea. Skeletal muscle biopsy analysis showed no mitochondrial abnormalities or respiratory chain dysfunction. Exome sequencing identified a* de novo* c.1651C>T (p.R551C) mutation in* STXBP1.* Although mitochondrial dysfunction has been reported in some individuals, our proband had only mild lactic acidemia and no skeletal muscle tissue evidence of mitochondrial disease pathology. Thus, mitochondrial dysfunction is not an obligate feature of* STXBP1* disease.

## 1. Introduction

Syntaxin Binding Protein 1 (*STXBP1*) or* MUNC18.1* was first implicated as a cause of pediatric epilepsy in 2008 [1]. Since then, the phenotypic range of heterozygous mutation carriers has expanded to include early-onset-epileptic encephalopathy with suppression-bursts (EESB), Ohtahara syndrome, West syndrome, unclassified infantile spasms, nonsyndromic intellectual disability, and nonepileptic movement disorders (summarized in [2]). Therefore, a consensus phenotype has been difficult to establish. Here, we report a 7-year-old boy who presented for diagnostic evaluation of intractable epilepsy, episodic ataxia, resting tremor, speech regression following a period of apparently normal early development, and concern for possible mitochondrial disease based on identification of mild lactic acidemia during an intercurrent illness.

## 2. Case Presentation

The proband presented to the Mitochondrial-Genetics Diagnostic Clinic at The Children's Hospital of Philadelphia at the age of 7 years for evaluation of seizures and developmental delay. He was a Caucasian male who was born at 38 weeks of gestation to a 23-year-old mother by repeat Cesarean section. The pregnancy was complicated by tobacco use and one episode of dehydration requiring intravenous fluids. His birth weight was 4200 g (>95th percentile) and length was 48 cm (50th percentile). He first came to medical attention at 5 months of age after an episode of staring, right arm jerking, and perioral cyanosis, which was diagnosed as idiopathic partial epilepsy. His electroencephalogram (EEG) at that time showed left temporal lobe seizures, for which he was begun on levetiracetam. He then developed a resting tremor at 8 months of age, which has persisted over time. He has also had episodic ataxia since late infancy that becomes exacerbated by viral illnesses. His epilepsy became refractory to medical treatment at 18 months of age despite multiple antiepileptic therapy that included phenobarbital, valproic acid, oxcarbazepine, and levetiracetam. He continued to have partial seizures and also developed atonic seizures around the age of 4 years, which prompted a repeat EEG at the age of 5 years that showed poor organization, background slowing, and multifocal sharps seen most frequently in the right and left frontal regions. At this time the ketogenic diet was initiated but was weaned at the age of 7 years when he continued to have frequent partial and atonic seizures and had no improvement in his ataxia. He has remained on valproic acid, oxcarbazepine, and levetiracetam since the age of 4 years. Brain magnetic resonance imaging (MRI) at the age of 4 years showed persistent nonmyelination in the anterior temporal lobe and frontal lobe terminal zones that was reported as potentially reflecting mildly delayed myelination but was otherwise normal.

His early developmental milestones were achieved on time, with walking at the age of 12 months and use of three words at 17 months. At 17 months, he was also able to play “peek-a-boo” but was not able to wave good-bye, could run with an ataxic “stomping” gait, and could transfer objects from hand to hand. He spoke several single words by 20 months, which he lost at the time of speech regression at 20 months. A vitamin B complex and levocarnitine were begun at the age of 20 months.

On review of systems, he had a clinically normal echocardiogram at the age of 5 years and normal audiology evaluation at the age of 3 years. He has had no growth, gastrointestinal, or renal issues. Dilated eye examination at the age of 3 years noted possible bilateral midperiphery retinal pigment mottling, although repeat ophthalmologic evaluation at 6 years of age was normal. Optical coherence tomography (OCT) testing at the age of 3 years demonstrated a bilaterally thickened ganglion cell layer in the perifovea. Although an electroretinogram (ERG) at the age of 3 years was suggestive of possible retinal dystrophy of both rods and cones, at follow-up evaluation it was felt that a retinal dystrophy was excluded based on normal dilated fundus examination and visual acuity testing. His visual acuity has always appeared to be normal. A three-generation pedigree was negative, with the exception of the proband's parents both having had febrile seizures but otherwise normal development and health. He has a healthy full brother and maternal half-sister. There is no recognized consanguinity.

Metabolic screening laboratory studies performed at the age of 20 months during a hospitalization for a viral illness showed elevations of blood lactate (3.2 mM, normal < 2.2 mM) and pyruvate (0.17 mM, normal < 0.14 mM), but normal alanine (493.5 *μ*mol/L, normal 119–523 *μ*mol/L). His lactate to pyruvate ratio was normal at 19 (normal range 10–20). Following his recovery, three weeks later, he had normal blood lactate (1.8 mM) and pyruvate (0.13 mM), as well as normal concurrent CSF amino acid analysis and pyruvate level. Free carnitine level at 22 months was 33 *μ*mol/L (age-adjusted normal range 31–51 *μ*mol/L) and total carnitine was 48 *μ*mol/L (age-adjusted normal range 45–66 *μ*mol/L). An acylcarnitine profile was reported as normal. Serum coenzyme Q levels also drawn at that time were normal at 0.79 mg/L (normal range 0.3–1.558 mg/L).

A quadriceps skeletal muscle biopsy was performed at the age of 3 years. Muscle histology and immunohistochemistry were normal. Electron transport chain enzyme activity analysis on frozen muscle was normal. Muscle mitochondrial DNA content analysis by quantitative PCR analysis in a clinical diagnostic laboratory was normal (135% relative to age and tissue-matched controls).

Upon examination at the age of 7 years, he weighed 24.2 kg (50th–75th percentile), was 125 cm tall (50th–75th percentile), and had a head circumference of 51.3 cm (25th–50th percentile). He was nondysmorphic except for medial eyebrow flare and thick eyelashes. He was able to walk with a wide-based gait but was nonverbal and unable to follow commands. His mental status was sleepy but alert and appeared interested in what was happening around him. He had minimal facial expression or reaction to conversation. He was generally calm but intermittently tried to bite the examiner. He was able to hold the tape measure but could not press the button. Pupils were equally round and reactive to light. He had a normal red reflex, extraocular movements were intact without nystagmus, and he was able to track an object. He had symmetric facial strength and smile. He had bilateral appendicular hypotonia with tight heel cords. His muscle bulk and strength was normal (5/5) throughout, but he was only able to stand with support due to resting tremor and ataxia. He was not able to perform finger-to-nose movements secondary to dysmetria. His deep tendon patellar reflexes were 2+ bilaterally. He had 2 to 3 beats of ankle clonus bilaterally.

### 2.1. Prior Clinical Diagnostic Testing

Screening for lysosomal storage disorders was unrevealing. N-Glycan and transferrin analysis, O-glycan analysis, urine oligosaccharide and glycan analysis, and urine creatine and guanidinoacetate analysis were all within the control range. Genome-wide single nucleotide polymorphism microarray analysis showed no chromosomal copy number alterations. Clinical diagnostic gene sequencing revealed no pathogenic mutations in* UBE3A*,* MECP2*,* FOXG1*,* PDHA1*,* ARX*,* TCF4*,* SCN1A*,* MEF2C*,* CDKL5*,* PPT1*, and* TPP1* or a panel of 101 nuclear genes implicated at that time in mitochondrial disease (Baylor mtDNA NGS May 2012). Whole mitochondrial DNA genome next generation sequencing in skeletal muscle identified a homoplasmic rare variant, m.14211C>T (p.V155L,* ND6*) that was considered to be a likely nonpathogenic polymorphism, as documented in the Human Mitochondrial Genome Database (mtDB).

### 2.2. Whole Exome Sequence Analysis

Whole blood DNA from the proband, his unaffected brother, and his mother was captured by Agilent SureSelect Human All Exon V4 kit and sequenced on a research basis in the Center for Applied Genomics at The Children's Hospital of Philadelphia on Illumina HiSeq 2500. A c.1651C>T, p.R551C mutation in* STXBP1* was identified that was not inherited from his mother and was absent in his healthy sibling. His unaffected maternal half-sister was subsequently confirmed to be negative for this mutation by Sanger sequencing. His father was unavailable for sequencing. However, as his father had no reported neurodevelopmental symptoms similar to the proband it remains likely that the* STXBP1 *mutation occurred* de novo* in the proband. The mutation was predicted to be pathogenic by SIFT, PolyPhen-2, LRT, and MutationTaster algorithms [3–6]. It was not seen in existing sequencing data from >2,000 exome datasets in our database or any public databases, including the 1,000 Genomes Project, NHLBI ESP6500SI, and the Exome Aggregation Consortium (ExAC) version 0.3 dataset. The same mutation had previously been reported in one other patient with an overlapping but nonidentical phenotype features [7]. Subsequently, the same mutation was confirmed on a clinical diagnostic basis both on the Greenwood Genetic Center Epilepsy Panel (August 2014) and on whole exome sequencing (WES). In addition, a single* CLN5* c.223T>C (p.W74R) variant as reported on clinical WES analysis suggests that he may be an asymptomatic carrier for autosomal recessive neuronal ceroid lipofuscinosis 5. No other pathogenic mutations that satisfactorily explained his phenotype were reported.

## 3. Discussion

*De novo STXBP1* disorders vary widely in their potential neurodevelopmental phenotypes. Indeed, some patients have shown significant improvement over time while others manifest a deteriorating clinical course. Approximately 50 patients have been reported in the literature, with no consistent features among all mutation carriers. In a helpful review by Barcia et al. nearly all of the 50 patients reviewed had some form of epilepsy, including early infantile epileptic encephalopathy with suppression-bursts (EESB), infantile spasms, focal epilepsy, and early-onset-epileptic encephalopathy [2]. Additional neurologic features have variably included dystonia, ataxia, hypotonia, stereotyped movements, and neuropathy [1, 2, 7–18]. A small minority of patients presented with isolated intellectual disability, although a wide range of intellectual ability can occur in this disorder from normal intelligence to profoundly disabled. Brain MRI findings have been similarly variable, including hypoplastic corpus callosum, cortical atrophy, and frontal hypoplasia. Our proband had many of the classic neurologic features of this disorder, although his brain MRI was largely normal.

There is one additional patient in the literature (Weckhuysen et al., patient F) with the same variant as our patient (c.1651C>T; p.R551C) with overlapping features [7]. The reported patient is a 20-year-old female with a normal exam at birth, who had seizure onset at 3 months that consisted of staring, blue lips, and irregular respirations. She then developed daily “epileptic spasms” controlled with valproic acid, but wringing movements of the torso and arms developed and were attributed to the valproic acid, which was subsequently stopped. The epileptic spasms returned but were controlled until the age of three when she developed focal seizures with staring and swallowing. She also has profound ID with ataxia and her most recent EEG at 18 years showed a slow background with multifocal 2.5–4 Hz sharp waves. The only testing reported on this patient is a normal karyotype and microarray, and no other clinical features, including those that would suggest abnormal retinal or mitochondrial functions, are reported. The epilepsy progression described in this patient is similar in onset to the patient reported here, although hers appeared to be more responsive to treatment. They both have the intellectual disability and ataxia findings, which are common in this disorder.

Two previous reports have suggested mitochondrial dysfunction may occur in* STXBP1* disease [8, 9]. Specifically, one older patient with a c.416C>T, p.Pro139Leu* de novo* dominant mutation and a second, younger patient with a c.585C>G, p.Tyr195X* de novo* dominant mutation in* STXBP1* showed muscle biopsy evidence of mitochondrial dysfunction in complexes I and IV, respectively. The older child presented with infantile epilepsy and developed Parkinsonian symptoms in her teens, with reduced complex I activity at 29% of the control mean (0.030 nmols NADH oxidized/min per unit of citrate synthase, control mean 0.104 ± 0.036). The younger child had infantile spasms and developmental delay, with follow-up only reported through 2 years of age. Mitochondrial disease was clinically suspected in the younger child based on a possibly elevated lactate peak in the basal ganglia on brain spectroscopy and mildly elevated blood lactate levels. He had marginally reduced mitochondrial complex IV levels at approximately 59% of the control mean (108 nmol/min/mg protein, normal 128–237 nmol/min/mg protein). However, neither of these changes in mitochondrial enzyme activities would meet classical criteria for mitochondrial respiratory chain dysfunction (below 20% of the control mean) [19]. Similarly, our* STXBP1* proband had normal mitochondrial enzyme activities and mitochondrial content. Thus,* STXBP1* disease does appear to have variable expressivity and mitochondrial respiratory chain dysfunction does not appear to be a feature of the disease.

STXBP1 is vital for neurotransmitter secretion through control of exocytosis, and* STXBP1-*null mice, while demonstrating normal brain development, die at birth due to their inability to breath as a result of impaired synaptic function [20]. As we detected no evidence of mitochondrial dysfunction in our case, it is likely that the suspected mitochondrial dysfunction previously reported may be attributed to environmental exposures or other modifying genetic factors, as mutations in* STXBP1* do not appear to contribute to this phenotype.

## 4. Conclusion

Here we report a 7-year-old Caucasian boy with a pathogenic missense mutation in* STXBP1* in whom mitochondrial dysfunction had been suspected based on having severe neurologic dysfunction (intractable mixed epilepsy, ataxia, tremor, appendicular hypertonia, clonus, dysmetria, speech regression, and delayed myelination) and ophthalmologic involvement (variably interpreted retinal pigment changes, abnormal OCT and ERG, and good visual function) with mild lactic acidemia at the time of intercurrent illness. However, extensive mitochondrial analyses in skeletal muscle were unrevealing and no pathogenic etiology was identified in known nuclear or mitochondrial genes that cause mitochondrial disease. Establishing both the correct diagnosis and accurate prognosis can be challenging in patients with complex neurologic phenotypes having variable multisystemic involvement. Exome sequencing is a valuable means to establish the underlying etiology in a wide range of neurologic conditions.
